# Shortcomings of ultrasound-guided fine needle aspiration in the axillary management of women with breast cancer

**DOI:** 10.1186/s12957-019-1753-y

**Published:** 2019-12-04

**Authors:** Michel Attieh, Faek Jamali, Ghina Berjawi, Mothana Saadeldine, Fouad Boulos

**Affiliations:** 1 Department of Pathology and Laboratory Medicine, American University of Beirut Medical Center, Riad El Solh 1107 2020, P.O. Box: 11-0236, Beirut, Lebanon; 2 Department of Surgery, American University of Beirut Medical Center, Beirut, Lebanon; 3 Department of Diagnostic Radiology, American University of Beirut Medical Center, Beirut, Lebanon

**Keywords:** Axillary lymph node dissection, Sentinel lymph node biopsy, Ultrasound-guided fine needle aspiration, Z0011 trial

## Abstract

**Background:**

Ultrasound, along with ultrasound-guided fine needle aspiration, is currently used for the axillary evaluation of breast cancer patients in order to identify candidates for axillary lymph node dissection. The aim of this study is to evaluate the accuracy of this tool in correctly identifying patients who may or may not benefit from axillary clearance in light of the ACOSOG Z0011 trial recommendations.

**Methods:**

One hundred one patients (65 with positive US-FNA with corresponding axillary lymph node dissection (ALND), and 36 with negative US-FNA with corresponding ALND/sentinel lymph node biopsy) were studied for the number of involved axillary lymph nodes, tumor clinicopathologic features, and axillary radiologic findings.

**Results:**

From the positive US-FNA group, 43% of patients had two or fewer positive lymph nodes upon ALND pathologic examination. In the US-FNA negative group, the negative predictive value for detecting axillary disease was 72.7%. With both groups combined, the sensitivity, specificity, PPV, and NPV of US-FNA for selecting patients based on axillary disease burden were 86%, 51.7%, 57%, and 83.3%, respectively.

**Conclusion:**

Based on Z0011 guidelines, US-FNA is not a reliable tool in triaging patients in need for ALND and leads to overtreatment of 43% patients when positive, while depriving a small but significant percentage of patients from necessary therapy, when negative.

## Introduction

Regional axillary lymph node metastasis from primary breast cancer has been accepted as part of the biology of breast cancer since the introduction of radical mastectomy by Halsted in 1894 [1]. Subsequently, the concept of sentinel lymph node excisional biopsy (SLNB) was introduced, substantiated by the convergence of lymphatics to the primary draining “sentinel” lymph node within the axilla. Given its association with a significantly lower risk of post-operative complications, most notably lymphedema, SLNB has become the most important diagnostic modality in identifying patients who could be spared full axillary lymph node dissection (ALND) [2].

In an effort to determine whether ALND was always justified following a positive SLNB, the Z0011 trial of the American College of Surgeons Oncology Group (ACSOG), which opened in 1999 and closed enrollment in 2004, assigned women with T1 or T2 breast cancer with positive SNLB to either completion ALND vs. no further surgical intervention or axillary radiation therapy. Trial results showed that regional recurrence was 0.9% with SLNB alone vs. 0.5% for ALND when the number of positive sentinel lymph nodes was either 1 or 2. The investigators concluded that patients with two or fewer positive sentinel lymph nodes do not benefit from complete axillary dissection due to the lack of a significantly different outcome between the two groups [3].

Axillary lymph node sampling by ultrasound-guided fine needle aspiration (US-FNA) has been recently advocated as a less invasive substitute to SNLB in patients with an ultrasonographically suspicious lymph node. A positive FNA would confirm axillary metastasis and normally lead to ALND. Based on current literature, however, a positive US-FNA, although associated with a higher axillary tumor burden than a positive sentinel lymph node, does not reliably predict the number of positive axillary lymph nodes. In a retrospective study of 234 patients, US-FNA of the axilla revealed a median of four positive axillary lymph nodes (range 1–30) in 158 patients with a malignant aspirate. Specifically, 43.9% (*n* = 69) of patients had 1–3 positive nodes [4].

Given this information and the recommendations of the Z0011 trial, we hypothesize that US-FNA of the axilla, though highly specific for detecting axillary lymph node metastasis, will result in a greater number of ALND than necessary, given its inability to reliably predict the number of involved lymph nodes (> 2 vs. ≤ 2). On the other hand, a negative US-FNA may not be sufficient to select patients who could be spared further axillary interventions. Our aim is to therefore assess the number of positive lymph nodes in ALND following either a positive US-FNA or a negative US-FNA through evaluation of subsequent ALND and/or SLNB, and to potentially identify predictive variables of axillary status, such as ultrasonographic findings, tumor size, tumor type, and tumor grade. Our findings may help predict which patients with a positive US-FNA would need—or could be spared—ALND according to the Z0011 trial guidelines, and whether a negative US-FNA could exempt patients from further invasive management.

## Materials and methods

### Patient selection and ultrasound findings

Following Institutional Review Board approval, a retrospective review of the Pathology/Cytology database at the American University of Beirut Medical Center (AUBMC), Beirut, Lebanon, was performed. Our target population was patients with clinically negative axillae, and positive US-FNA with a corresponding ALND, or negative US-FNA with corresponding SLNB/ALND. Patients with a positive FNA and negative ALND who received neoadjuvant treatment were excluded from the analysis. Sixty-five patients with positive US-FNA and thirty-six patients with negative US-FNA were retrieved.

The ultrasounds and FNAs were performed by specialized interventional radiologists in the field of breast cancer radiology with at least 10 years of experience. Positive ultrasound findings included single or multiple enlarged lymph nodes, focally or diffusely thickened cortex (more than 3 mm), abnormal lobulation, calcifications, and Doppler signals.

Suspicious lymph nodes, with at least one of the above criteria, were selected for aspiration. The nodes with thick cortices were aspirated in the thickest areas, and the puncture site was directed specifically at the thickest cortex, as detailed by Mainiero et al. [5].

### Clinicopathologic characteristics

Clinicopathologic features including age, gender, tumor histology, grade, size, number of lymph nodes involved, size of LN metastasis, and prior neoadjuvant chemotherapy were recorded.

### Statistical analysis

Numerical variables were described by their mean, median, and standard deviation, whereas categorical variables were described by their relative frequencies and counts. Fisher’s exact test was used to compare nominal data in various groups. Mann-Whitney *U* test was used for nonparametric data to test differences between the number of positive lymph nodes (cutoff limit of two lymph nodes) with respect to age and size of the primary tumor. Similarly, Mann-Whitney *U* test was used to test differences between the size of metastasis (cutoff limit of 0.9 cm) with respect to age and size of the primary tumor. The diagnostic accuracy was evaluated with sensitivity, specificity, and positive predictive value (PPV) and negative predictive values (NPV). The receiver operating characteristics (ROC) curves and the respective areas under the curves (AUC) were also calculated for the different parameters. Results were expressed with a 95% confidence interval (CI). Binary logistic regression was utilized to calculate any difference between U/S FNA findings and the number of involved lymph nodes. Two-sided *P* values of less than 0.05 were considered to indicate statistical significance. Computational analyses were performed using SPSS Statistical Package for Social Sciences version 25.0 (Chicago, IL, USA) and via AnalystSoft, StatPlus:mac statistical analysis program for Mac OS. See http://www.analystsoft.com/en/.

## Results

Clinicopathologic characteristics, including age distribution, type of cancer, tumor size, and tumor grade in both US-FNA positive and negative groups, are summarized in Table 1. Ultrasound findings for both groups are outlined in Table 2.

**Table 1:** Clinicopathologic features of patients with positive vs. negative axillary US-FNA

Variables	Positive US-FNA [*N*(%)]	Negative US-FNA [*N*(%)]
Average age	52.53 ± 13.2	53 ± 11.1
Tumor type		
IDC	39/65 (60)	28/36 (77.8)
IDC (residual)	13/65 (20)	0/36 (0)
IDC-DCIS	0/65 (0)	1/36 (2.8)
IDC/ILC	0/65 (0)	3/36 (8.3)
ILC	2/65 (3.1)	2/36 (5.6)
ILC (residual)	1/65 (1.5)	0/36 (0)
DCIS	1/65 (1.5)	0/36 (0)
DCIS (residual)	1/65 (1.5)	0/36 (0)
Adenosquamous	0/65 (0)	1/36 (2.8)
IMC with neuroendocrine features	0/65 (0)	1/36 (2.8)
No residual	6/65 (9.2)	0/36 (0)
No tumor	1/65 (1.5)	0/36 (0)
NA	1/65 (1.5)	0/36 (0)
Modified SBR grade		
(3/3)	23/65 (35.4)	19/36 (52.8)
(2/3)	23/65 (35.4)	12/36 (33.3)
(1/3)	4/65 (6.2)	3/36 (8.3)
NA	15/65 (23)	2/36 (5.6)
Size	Mean = 3.4 +/- 3.3 cm	Mean = 2.3 ± 1.5 cm
	Median = 2.5 cm	Median = 2.0 cm
Neoadjuvant therapy		
None	43/65 (66.2)	33/36 (91.7)
Yes	21/65 (32.3)	3/36 (8.3)
NA	1/65 (1.5)	0/36 (0)

*US-FNA* ultrasound-guided fine needle aspiration, *IDC* invasive ductal carcinoma, *ILC* invasive lobular carcinoma, *DCIS* ductal carcinoma in situ, *IMC* invasive mammary carcinoma, *SBR* Scarff-Bloom-Richardson, *NA* not available

**Table 2:** Ultrasound findings of positive and negative US-FNA cases

Ultrasound findings	Positive US-FNA number (%)	Negative US-FNA number (%)
Enlarged lymph node	34/65 (52.3)	2/36 (5.6)
Diffusely thick cortex	12/65 (18.6)	22/36 (61.1)
Focally thick cortex	0/65 (0)	6/36 (16.7)
Enlarged lymph node + thick cortex	6/65 (9.2)	1/36 (2.8)
Multiple enlarged lymph nodes + thick cortex	1/65 (1.5)	2/36 (5.6)
Calcification	1/65 (1.5)	0/36 (0)
Multiple enlarged lymph nodes + calcification	1/65 (1.5)	0/36 (0)
Normal	2/65 (3.1)	0/36 (0)
NA	8/65 (12.3)	3/36 (8.3)

*US-FNA* ultrasound-guided fine needle aspiration, *NA* not available

Statistical analysis was performed to assess the significance of different variables in both groups. In the US-FNA positive group, a significant correlation was identified between the number of involved lymph nodes (cutoff limit of two lymph nodes) and primary tumor size, and between the number of involved lymph nodes and metastatic deposit size (Table 3). At a primary tumor size of 2.3 cm, sensitivity and specificity were both 70%. Of the cases with ≤ 2 involved lymph nodes, 3/28 were ≥ 3 cm; however, in cases with > 2 involved lymph nodes, 2/37 were ≤ 1 cm. The ROC curve showed high specificity (94.7%) for predicting three or more positive axillary lymph nodes when the primary tumor is more than 5.2 cm in diameter (namely T3 lesions). No such statistically significant correlations were found between the different clinicopathologic variables in the US-FNA negative group (Table 4). There was no significant correlation between axillary lymph node US findings and the number of involved lymph nodes (≤ 2 or > 2), or the size of metastatic deposits (Table 5).

**Table 3:** Correlation between different clinicopathologic and radiologic parameters in the US-FNA positive group

Variable	*P* value
Number of involved LNs vs. age	*P* = 0.832
Number of involved LNs vs tumor size	*P* = 0.002**
Size of LN metastasis vs age	*P* = 0.046**
Size of LN metastasis vs. tumor size	*P* = 0.22
Number of involved LNs vs. size of LN metastasis	*P* = 0.034*

*US* ultrasound, *LN* lymph node

*Fisher’s exact test (chi-squared test)

**Via Mann-Whitney test

**Table 4:** Correlation between the different clinicopathologic variables in the US-FNA negative group

Variables	*P* value
Number of involved LNs vs. age	*P* = 0.285*
Number of involved LNs vs. tumor size	*P* = 0.766 **
Number of involved LNs vs. grade	*P* = 0.908*
Number of involved LNs vs. neoadjuvant therapy	*P* = 0.336*

*LN* lymph node

*Fisher’s exact test (chi-squared test)

**Mann-Whitney test

**Table 5:** Correlation of ultrasound findings with the number of involved lymph nodes and with the size of lymph node metastasis

US findings vs. number of involved LNs	*P* = 0.694
US findings vs. size of LN metastasis	*P* = 0.254

*US* ultrasound, *LN* lymph node

In the US-FNA positive group, cases with ≤ 2 involved lymph nodes constituted 43% (28/65) of the sample size, while cases with > 2 involved lymph nodes represented 57% (37/65) of the cases, with a median of seven positive lymph nodes (range 3–36 out of 10–52). In the US-FNA negative group, lymph node involvement was identified in 33.3% (12/36) of the cases with a median metastatic deposit size of 0.9 cm. Extranodal extension was present in 33.3% (4/12) of the involved lymph nodes. In order to address the utility of US-FNA, we calculated its sensitivity, specificity, PPV, and NPV in light of the Z0011 trial recommendations. The negative predictive value (NPV) was 66.7% with a 33.3% corresponding rate of positive lymph nodes. When micrometastasis was excluded (*n* = 3), the NPV became 72.7%, (i.e., 27.3% of patients with negative US-FNA had positive lymph nodes). Fifty percent (6/12) of the positive axillae contained more than two positive lymph nodes (16.6% of all cases). The negative predictive value of US-FNA for identifying axillae with more than two positive lymph nodes was therefore 83.3% (30/36). Assuming that there need to be more than two positive lymph nodes for an US-FNA to be considered positive and to warrant ALND, US-FNA would have a sensitivity of 86%, a specificity of 51.7%, a PPV of 57%, and a NPV of 83.3%.

## Discussion

According to the National Comprehensive Cancer Network (NCCN) [6] and the British National Institute of Clinical Excellence [7], the recommendations for preoperative axillary staging of patients with invasive breast cancer requires performing an axillary US in clinically node-negative patients, to be followed by an US-FNA for ultrasonographically suspicious axillary lymph nodes or in clinically node-positive patients.

There is little doubt that US-FNA is a reasonably sensitive and highly specific minimally invasive procedure for identifying axillary metastasis in breast cancer patients. Cools-Lartigue and Meterissian showed that the sensitivity and specificity of axillary US-FNA in patients with invasive breast cancer range from 5.7 to 62.9% and 95.5 to 100%, respectively [8]. Ultrasound typically utilizes characteristics such as a thick cortex (> 3 mm) as a feature suggestive of an involved lymph node with an accuracy rate > 70% (range 70–90%) [8–10]. Reliance on cortical thickness and other sonographic findings has significantly improved the positive predictive value of US-FNA, reaching 97.1% (66/68) and 100% (30/30) in some reports [11, 12]. Conversely, the negative predictive value showed mixed results, with one study quoting a value of 69.8% [12] and another reporting a value of 89.1% [13]. Although the false-negative rate was reported by Leenders et al. to reach 28.1% [14], a positive/suspicious US, irrespective of the US-FNA cytology findings, led to an ALND in Rattay et al.’s study based on a post-test nodal involvement probability of 78% [15]. The arguments in favor of initial assessment by US-FNA over SLNB also abound, and do stand to reason. Comparative analysis between US-FNA and SLNB showed that performing US-FNA resulted in a reduction in SLNB reaching 40% [16, 17]. The rationale here is one of avoiding an additional surgical procedure, and reducing the cost by up to 20% [15].

Based on the above and similar research, the NCCN 2014 guidelines for the workup of stages I, IIA, IIB, and IIIA in patients with a positive US-FNA are currently to proceed towards level I/II axillary dissection. This, however, advocates a blanket approach of axillary clearance for all patients with any degree of axillary metastasis. Namely, it does not address the Z0011 trial findings that have identified a subgroup of patients with only two or less involved lymph nodes and an overall survival similar to patients with completion ALND [18].

The questions we have tried to answer in this study and in light of the Z0011 trial are the following: (1) how often do patients with a positive US-FNA end up with two or fewer positive axillary lymph nodes upon ALND, (2) whether US findings and tumor characteristics can help identify patients who are likely to have > 2 positive axillary lymph nodes and who indeed would benefit from ALND, and (3) whether a negative US-FNA could spare patients from SLNB and ALND.

First, and as presented above, US-guided axillary FNA was not able, in our series, to classify patients according to the number of involved lymph nodes (> 2 or ≤ 2). Although patients with a positive US-FNA do have significantly more positive lymph nodes upon axillary dissection than those with SLNB, as shown by van Wely and colleagues in their comprehensive meta-analysis [19], we and others [4] have shown that close to half of patients with a positive US-FNA end up with two or less positive axillary lymph nodes following dissection. Moreover, a non-negligible proportion of these patients have a low-volume metastatic nodal disease (< 5 mm) [20], and performing an ALND on such patients, irrespective of the Z0011 trial, remains controversial [20, 21]. As stated by Lloyd et al., axillary ultrasound did detect a higher axillary tumor burden than in patients who underwent sentinel lymph node biopsy; however, 40% of the axillary ultrasound group had two or fewer lymph nodes with macrometastasis after axillary lymph node dissection and hence were subject to overtreatment [22]. Moreover, 78% of women with invasive breast carcinoma measuring 2 cm or less who also had one abnormal lymph node on axillary ultrasound were shown to have two or fewer involved nodes upon axillary lymph node dissection and would have benefited from sentinel lymph node biopsy and avoided axillary surgery, as shown by Puri et al. [23].

Second, we were unable to predict the number of positive axillary lymph nodes based on sonographic characteristics, and only marginally based on primary tumor size. Although Moore et al. were able to correlate specific US findings with an overall axillary stage (increased cortical thickness = N1, loss of fatty hilum/increased vascularity/abnormal shape = N2–3), no such correlation could be obtained in our series (*P* > 0.05). With respect to tumor characteristics, a statistically significant correlation was only found between primary tumor size and the number of involved lymph nodes. A PPV of 88% and a specificity of 95% for having > 2 positive axillary lymph nodes could only be obtained with primary tumor size > 5.2 cm. This finding is of marginal significance given the standard practice of ALND in patients with T3 disease irrespective of preoperative axillary staging, and the exclusion of T3 tumors from the Z0011 trial [24]. Furthermore, Jain et al. reported no significant correlation between primary tumor features (cutoff size of 2 cm) and US-FNA status with final nodal pathology [25]

Another limitation of US-FNA is that metastatic nodal size, which cannot be predicted by US or by FNA, was shown to exhibit a significant correlation with the number of involved lymph nodes. Corroborative studies on metastatic nodal size by Cedolini et al. determined an exponential relationship between the metastatic nodal size and number of involved lymph nodes, with micrometastasis associated with one positive non-sentinel lymph node, macrometastasis associated with 2.65 positive non-sentinel lymph nodes, and peri-capsular invasion with 9.88 positive non-sentinel lymph nodes [26]. In the Z0011 trial, approximately 45% of the patients who received SLNB alone had micrometastatic disease, implying that loco-regional control by SLNB is in part due to the size of the metastatic tumor deposit [27]. Similarly, Gutierrez et al. identified a significant correlation between sentinel lymph nodes showing isolated tumor cells vs. micrometastasis, and the extent of non-sentinel node positivity upon axillary clearance [28].

Third, we found that axillary FNA has an NPV of 72.7% for any degree of axillary metastasis. When taking into consideration the Z0011 guidelines for axillary clearance, the NPV of US-FNA becomes 83.3%. This implies that, if axillary US were to be used as the sole diagnostic tool to establish the absence of significant axillary burden, 16.7% of patients with a negative axillary US would have significant axillary disease and would be deprived of the necessary therapeutic axillary intervention.

## Conclusion

US-FNA carries a low positive predictive value of 57% for identifying patients who need an axillary clearance. On the other hand, 16.7% of patients with a negative US-FNA end up with more than two positive lymph nodes and an under-representation of the extent of their axillary disease. This ultimately renders US-FNA suboptimal for selecting patients likely to benefit from ALND as this technique offers a qualitative assessment of a parameter that requires quantitative evaluation.

As Dr. Giuliano, the primary investigator of the Z0011 trial, cautiously stated, irrespective of the Z0011 findings, ALND remains the standard of care for patients with axillary lymph node metastasis, and physicians should consider all pertinent elements in the final management decisions related to their patients’ breast cancer [29]. However, in an era where more conservative surgical approaches to breast cancer have successfully replaced more radical methods of treatment, we believe that performing blanket ALND based on a purely qualitative method of axillary staging is very likely to prove excessive. We believe that US-FNA, regardless of the outcome, may not represent an ideal tool in assessing preoperative axillary status, as it goes against the general modern trend of conservative and personalized treatment, away from potentially debilitating surgical interventions. However, a positive US-FNA in the context of a primary tumor more than 5 cm in diameter is highly predictive of three or more positive lymph nodes and justifies ALND without SLNB. We foresee that the only benefit of the minimally invasive US-guided FNA modality is to identify patients in need of additional axillary treatment, such as radiation therapy as demonstrated by the AMAROS trial, sparing patients the side effects of axillary surgery be it ALND or sentinel lymph node biopsy [30–32]. Until this is substantiated by randomized controlled clinical trials, we suggest replacing US-FNA with the more accurate SLNB, except in the assessment of candidates for neoadjuvant treatment where minimally invasive axillary staging would be appropriate. Until minimally invasive (or non-invasive) methods of axillary staging become more quantitative (such as US-FNA of multiple lymph nodes), we believe clinicians should carefully review the use of US-FNA as a means to decide on a patient’s axillary clearance, without garnering more information from a replacement, or at least a complimentary sentinel lymph node biopsy.

## Data Availability

The datasets used and/or analyzed during the current study are available from the corresponding author on reasonable request.

## References

[1] Halsted W (1895). The results of operations for the cure of cancer of the breast performed at the Johns Hopkins Hospital from June 1889 to January 1894. Johns Hopkins Hosp Bull.

[2] Habal N, Giuliano AE, Morton DL (2001). The use of sentinel lymphadenectomy to identify candidates for postoperative adjuvant therapy of melanoma and breast cancer. Semin Oncol.

[3] Giuliano A. E., McCall L. M., Beitsch P. D., Whitworth P. W., Morrow M., Blumencranz P. W., Leitch A. M., Saha S., Hunt K., Ballman K. V. (2010). ACOSOG Z0011: A randomized trial of axillary node dissection in women with clinical T1-2 N0 M0 breast cancer who have a positive sentinel node. Journal of Clinical Oncology.

[4] Van Wely B, De Wilt J, Schout P, Kooistra B, Wauters C, Venderinck D (2013). Ultrasound-guided fine-needle aspiration of suspicious nodes in breast cancer patients; selecting patients with extensive nodal involvement. Breast Cancer Res Treat.

[5] Mainiero MB, Cinelli CM, Koelliker SL, Graves TA, Chung MA (2010). Axillary ultrasound and fine-needle aspiration in the preoperative evaluation of the breast cancer patient: an algorithm based on tumor size and lymph node appearance. AJR Am J Roentgenol.

[6] Gradishar WJ, Anderson BO, Balassanian R, Blair SL, Burstein HJ, Cyr A (2017). NCCN guidelines insights: breast cancer, version 1.2017. J Natl Compr Cancer Netw.

[7] Harnett A, Smallwood J, Titshall V, Champion A (2009). Diagnosis and treatment of early breast cancer, including locally advanced disease—summary of NICE guidance. Bmj..

[8] Cools-Lartigue J, Meterissian S (2012). Accuracy of axillary ultrasound in the diagnosis of nodal metastasis in invasive breast cancer: a review. World J Surg.

[9] Cools-Lartigue J, Sinclair A, Trabulsi N, Meguerditchian A, Mesurolle B, Fuhrer R (2013). Preoperative axillary ultrasound and fine-needle aspiration biopsy in the diagnosis of axillary metastases in patients with breast cancer: predictors of accuracy and future implications. Ann Surg Oncol.

[10] Elmore LC, Appleton CM, Zhou G, Margenthaler JA (2013). Axillary ultrasound in patients with clinically node-negative breast cancer: which features are predictive of disease?. J Surg Res.

[11] Nath J, Sami N, Massey J, Donnelly J, Corder A (2013). Selection for axillary clearance in breast cancer (ultrasound negative, sentinel node positive patients have low rates of further metastases). Eur J Surg Oncol.

[12] Hu X, Zhou X, Yang H, Wei W, Jiang Y, Liu J (2018). Axillary ultrasound and fine needle aspiration biopsy in the preoperative diagnosis of axillary metastases in early-stage breast cancer. Oncol Lett.

[13] Ameri Bita, Kanesa-Thasan Riti, Abu-Khalaf Maysa M., Berger Adam C., Eisenberg Tara, Lazar Melissa A., Sevrukov Alexander, Tsangaris Theodore N., Wasti Nylah, Wilkes Annina, Willis Alliric Isaac, Zorn Lisa, Hsu Elizabeth (2017). Negative predictive value of ipsilateral axillary ultrasound in the pre-operative assessment of invasive breast cancer. Journal of Clinical Oncology.

[14] Leenders M, Broeders M, Croese C, Richir M, Go H, Langenhorst B (2012). Ultrasound and fine needle aspiration cytology of axillary lymph nodes in breast cancer. To do or not to do?. Breast..

[15] Rattay T, Muttalib M, Khalifa E, Duncan A, Parker S (2012). Clinical utility of routine pre-operative axillary ultrasound and fine needle aspiration cytology in patient selection for sentinel lymph node biopsy. Breast..

[16] Davis JT, Brill YM, Simmons S, Sachleben BC, Cibull ML, McGrath P (2006). Ultrasound-guided fine-needle aspiration of clinically negative lymph nodes versus sentinel node mapping in patients at high risk for axillary metastasis. Ann Surg Oncol.

[17] van Rijk MC, Deurloo EE, Nieweg OE, Gilhuijs KG, Peterse JL, Rutgers EJ (2006). Ultrasonography and fine-needle aspiration cytology can spare breast cancer patients unnecessary sentinel lymph node biopsy. Ann Surg Oncol.

[18] Yi M, Kuerer HM, Mittendorf EA, Hwang RF, Caudle AS, Bedrosian I (2013). Impact of the American College of Surgeons Oncology Group Z0011 criteria applied to a contemporary patient population. J Am Coll Surg.

[19] Van Wely B, De Wilt J, Francissen C, Teerenstra S, Strobbe L (2015). Meta-analysis of ultrasound-guided biopsy of suspicious axillary lymph nodes in the selection of patients with extensive axillary tumour burden in breast cancer. Br J Surg.

[20] Stachs A, Göde K, Hartmann S, Stengel B, Nierling U, Dieterich M (2013). Accuracy of axillary ultrasound in preoperative nodal staging of breast cancer-size of metastases as limiting factor. SpringerPlus..

[21] Fournier K, Schiller A, Perry RR, Laronga C (2004). Micrometastasis in the sentinel lymph node of breast cancer does not mandate completion axillary dissection. Ann Surg.

[22] Lloyd P, Theophilidou E, Newcombe RG, Pugh L, Goyal A (2017). Axillary tumour burden in women with a fine-needle aspiration/core biopsy-proven positive node on ultrasonography compared to women with a positive sentinel node. Br J Surg.

[23] Puri S, Sharma N, Newcombe RG, Kaushik M, Al-Attar M, Pascaline S (2018). Axillary tumour burden in women with one abnormal node on ultrasound compared to women with multiple abnormal nodes. Clin Radiol.

[24] Lyman GH, Temin S, Edge SB, Newman LA, Turner RR, Weaver DL (2014). Sentinel lymph node biopsy for patients with early-stage breast cancer: American Society of Clinical Oncology clinical practice guideline update. J Clin Oncol.

[25] Jain A, Haisfield-Wolfe ME, Lange J, Ahuja N, Khouri N, Tsangaris T (2008). The role of ultrasound-guided fine-needle aspiration of axillary nodes in the staging of breast cancer. Ann Surg Oncol.

[26] Cedolini C, Bertozzi S, Seriau L, Londero AP, Concina S, Cattin F (2014). Eight-year experience with the intraoperative frozen section examination of sentinel lymph node biopsy for breast cancer in a North-Italian university center. Int J Clin Exp Pathol.

[27] Giuliano AE, McCall L, Beitsch P, Whitworth PW, Blumencranz P, Leitch AM (2010). Locoregional recurrence after sentinel lymph node dissection with or without axillary dissection in patients with sentinel lymph node metastases: the American College of Surgeons Oncology Group Z0011 randomized trial. Ann Surg.

[28] Gutierrez J, Dunn D, Bretzke M, Johnson E, O’Leary J, Stoller D (2011). Pathologic evaluation of axillary dissection specimens following unexpected identification of tumor within sentinel lymph nodes. Arch Pathol Lab Med.

[29] Giuliano AE, Morrow M, Duggal S, Julian TB (2012). Should ACOSOG Z0011 change practice with respect to axillary lymph node dissection for a positive sentinel lymph node biopsy in breast cancer?. Clin Exp Metastasis.

[30] Donker M, van Tienhoven G, Straver ME, Meijnen P, van de Velde CJH, Mansel RE (2014). Radiotherapy or surgery of the axilla after a positive sentinel node in breast cancer (EORTC 10981-22023 AMAROS): a randomised, multicentre, open-label, phase 3 non-inferiority trial. Lancet Oncol.

[31] Soares EWS, Nagai HM, Bredt LC, da Cunha AD, Andrade RJ, Soares GVS (2014). Morbidity after conventional dissection of axillary lymph nodes in breast cancer patients. World J Surg Oncol.

[32] Purushotham AD, Upponi S, Klevesath MB, Bobrow L, Millar K, Myles JP (2005). Morbidity after sentinel lymph node biopsy in primary breast cancer: results from a randomized controlled trial. J Clin Oncol.

